# Silencing of casein kinase 1 delta reduces migration and metastasis of triple negative breast cancer cells

**DOI:** 10.18632/oncotarget.25738

**Published:** 2018-07-20

**Authors:** Isabelle Bar, Ahmad Merhi, Lionel Larbanoix, Manuel Constant, Sandy Haussy, Sophie Laurent, Jean-Luc Canon, Paul Delrée

**Affiliations:** ^1^ Laboratory of Translational Oncology, Institute of Pathology and Genetics/Grand Hôpital de Charleroi, Gosselies 6041, Belgium; ^2^ IPG BioBank, Institute of Pathology and Genetics, Gosselies 6041, Belgium; ^3^ Center for Microscopy and Molecular Imaging (CMMI), Université de Mons (UMONS), Charleroi 6041, Belgium; ^4^ Department of General, Organic and Biomedical Chemistry, NMR and Molecular Imaging Laboratory, Université de Mons (UMONS), Mons 7000, Belgium; ^5^ Service of Oncology-Hematology, Grand Hôpital de Charleroi, Charleroi 6000, Belgium; ^6^ Department of Pathology, Institute of Pathology and Genetics, Gosselies 6041, Belgium

**Keywords:** triple-negative breast cancer, migration, metastasis, tight junction, casein kinase 1 delta

## Abstract

The casein kinase 1 delta (CSNK1D) is a conserved serine/threonine protein kinase that regulates diverse cellular processes including cell cycle progression, circadian rhythm, and neurite outgrowth. Aberrant expression of CSNK1D is described in several cancer types including breast cancer, where it is amplified in about 30% of triple negative breast (TNBC). Here, we have investigated the function of CSNK1D in triple negative cancer cell migration and metastasis.

By using immunohistochemistry and *in situ* hybridization, we found that CNSK1D is highly expressed in primary tumor cells and in tumor cells invading lymphatic nodes compared to non-metastatic tumors. *In vitro*, knock-down of *CSNK1D* expression with specific shRNAs in the breast cancer cell line MDA-MB-231 markedly inhibited cancer cell proliferation, invasion and migration and affected the expression of the tight junction proteins claudin 1, occludin and the junction adhesion molecule A. *In vivo*, the inactivation of *CSNK1D* reduced lung metastasis in MDA-MB-231 breast cancer xenografts.

Altogether, our results indicate that the downregulation of CSNK1D expression inhibits the proliferation and reduces the migration and the metastasis of breast cancer cells. As numerous inhibitors of CSNK1D are currently under development, this might represent an attractive therapeutic target for the treatment of TNBC.

## INTRODUCTION

The triple-negative breast cancer subtype (TNBC) occurs in about 15% of breast cancer cases and is defined by the lack of oestrogen receptor, progesterone receptor and *HER2* gene amplification. This cancer is very aggressive with a poorer outcome compared to other breast cancer subtypes [1]. The risk of distant metastasis is higher in triple negative breast cancer relative to other molecular subtypes [2, 3]. Furthermore, treatment of patients with TNBC is more challenging due to the heterogeneity of the disease and the absence of well-defined molecular targets.

In a previous work, we have identified a genomic region amplified in some triple negative breast cancers [4]. One of the genes localized in this region is the casein kinase 1 delta gene (CSNK1D). CSNK1D is a member of a family of serine/threonine protein kinase that consists of six human isoforms (α, δ, ε, γ1, γ2 and γ3) [5]. These kinases are involved in several signaling pathways including Wnt, Hedgehog and the Hippo signaling, and can regulate numerous cellular processes like membrane trafficking, cytoskeleton maintenance, DNA replication, DNA damage response, RNA metabolism and circadian rhythm [5–7].

An alteration in the expression of some of these casein kinase proteins has been detected in cancer pathologies [8]. In melanoma, a decrease in the expression of casein kinase 1 alpha (CSNK1A) promotes growth and metastasis by activating the Wnt/β-catenin signaling pathway [9, 10]. A role for CSNK1A has also been also described in myeloma and lymphoma [11, 12].

On the other hand, high casein kinase 1 epsilon (CSNK1E) is correlated with better prognosis in breast cancer [13] and the loss of CSNK1E is associated with poorer prognosis in patients with low stage oral cancer and in patients with hepatocellular carcinoma [14, 15]. Furthermore, mutations in CSNK1E decrease cell adhesion and promote cell migration in breast cancer [16].

CSNK1D is overexpressed in cell lines and tissues of different origins like cancers of brain, prostate, colon, ovary, and hematopoietic and lymphatic system [7]. High level of CSNK1D expression has also been detected in pancreatic cancer [17]. Aberrant expression of CSNK1D is described in cervical squamous cell carcinoma and the increased expression is associated with deep cervical stromal invasion [18]. Furthermore, decreased CSNK1D expression is associated with prolonged survival in patients with colorectal cancer [19].

We previously reported that CSNK1D, localized in the 17q25.3 genomic region, is frequently amplified in triple negative breast cancer, particularly in *BRCA1* mutated breast cancer, and the increased copy number correlates with increased CSNK1D mRNA [4]. The role of CSNK1D overexpression in breast cancer remained poorly investigated until recently. Rosenberg *et al*, showed that the pharmacological inhibition of CSNK1D affects breast tumor growth in mouse orthotopic xenografts and in patient-derived xenografts [20]. Although CSNK1D protein seems to play a crucial role in the growth of the triple negative breast tumors, its role in migration and metastasis is unknown.

In this study, we provide evidences that CSNK1D plays a role in breast cancer cell migration and metastasis. Using *in situ* hybridization and immunohistochemistry, we confirm that the CSNK1D protein is highly expressed in primary breast tumor and matched metastatic lymph nodes. We show that the depletion of CSNK1D using shRNA mediated knock-down or pharmaceutical inhibition of CSNK1D significantly reduces the migration and invasion of MDA-MB-231 breast cancer cells *in vitro*. We further show that knock-down of CSNK1D increases the expression of tight junction proteins. Finally, we show that depletion of CSNK1D suppresses growth of human breast cancer xenografts and inhibits their metastatic potential *in vivo*. Together, our findings highlight an important role for CSNK1D in migration and metastasis of TBNC cells.

## RESULTS

### CSNK1D is overexpressed by tumor cells in primary tumor and metastatic lymph nodes

**Figure 1:**
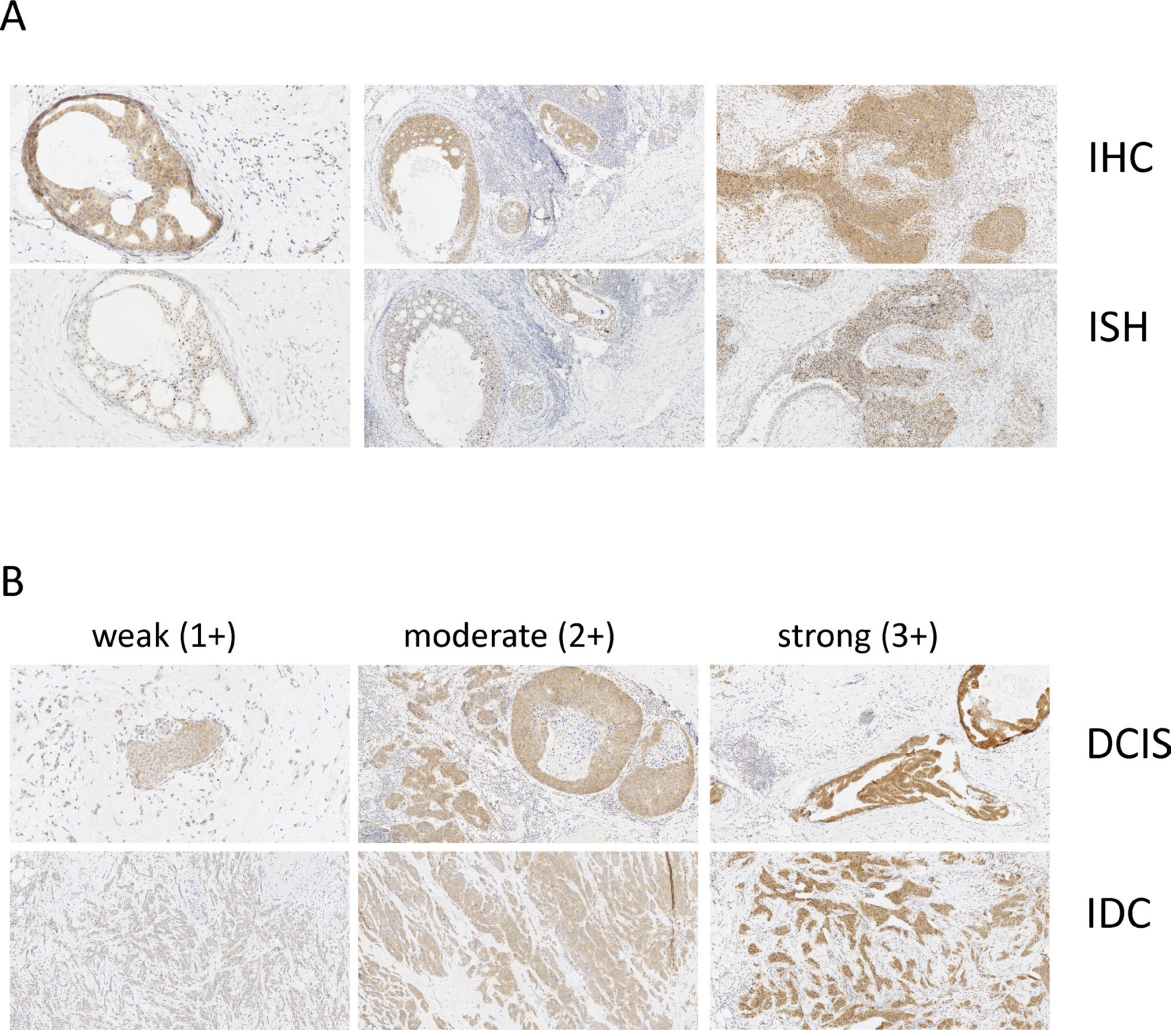
Validation of CSNK1D IHC (**A**) Serial sections of breast tumor were stained with CSNK1D antibody (upper line) or CSNK1D ISH probe (bottom line). Magnification 10×. (**B**) Illustration of weak (score 1+), moderate (score 2+) and strong (score 3+) CSNK1D immunoreactivity. Magnification 10×.

To explore the relevance of CSNK1D expression in breast cancer, we analyzed CSNK1D expression in primary tumors and matched node metastasis. In a first step, the specificity of the antibody was validated by comparing the detection of CSNK1D by IHC and ISH on serial sections of breast tumor. Similar results were obtained by ISH and IHC, confirming the specificity of the immunohistochemical detection of CSNK1D (Figure 1A). In addition, weak to strong CSNK1D immunoreactivity was observed in ductal carcinoma *in situ* (DCIS) and in invasive breast tumors (IDC) (Figure 1B).

**Figure 2:**
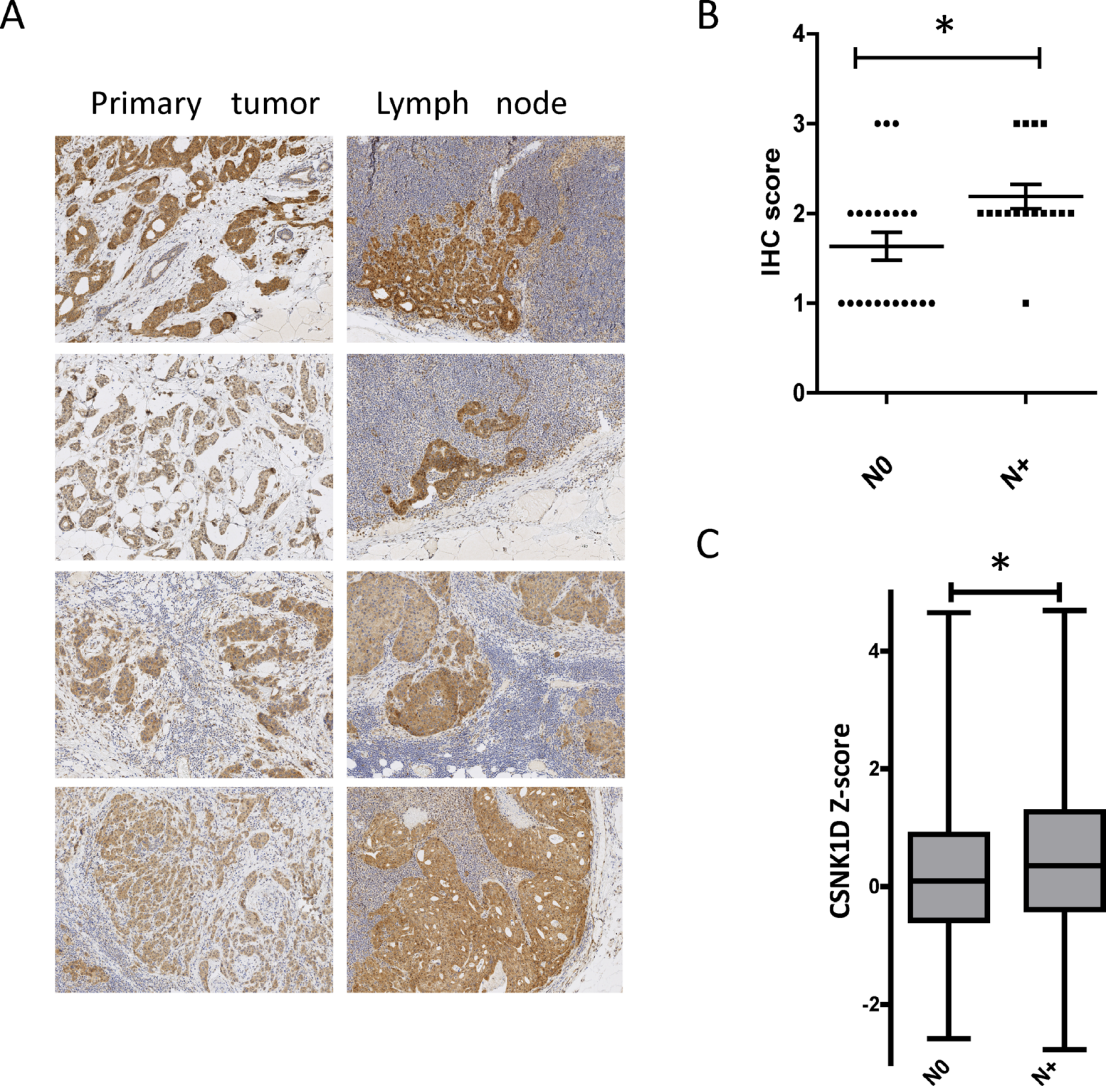
Expression of CSNK1D in primary tumors and metastatic lymph nodes (**A**) Representative IHC staining of CSNK1D in a primary lesion (left) and the corresponding lymph nodes (right). (**B**) Scoring of CSNK1D IHC in primary tumors without (N0) or with (N+) lymph node metastasis (error bars represent mean + SEM, Mann–Whitney test: ^*^=*p* < 0.05). (**C**) Analysis of CSNK1D mRNA expression in TCGA data (breast invasive carcinoma project, Nature 2012). Whiskers: minimum and maximum. bar: median. Statistical analysis: Mann–Whitney test: ^*^=*p* < 0.05.

In a second step, the expression level of CSNK1D was assessed by IHC in 38 primary tumors (9 LumA, 10 LumB, 7 HER2 and 12 TN) and 13 corresponding metastatic lymph nodes (3 LumA, 3 LumB, 3 HER2 and 4 TNBC) (Table 1). Strong cytoplasmic expression in the tumor cells invading lymph nodes was observed at a level comparable or higher than the expression observed in the primary tumor (Figure 2A).

**Table 1:** Patient and tumor characteristics

	Lum A	Lum B	HER2^+^	TN
Age, mean (min-max)	58 (42–71)	58 (40–78)	66 (53–88)	48 (34–67)
Grade, n (%)				
I	3 (33)	0 (0)	0 (0)	0 (0)
II	6 (67)	5 (50)	2 (28)	2 (17)
III	0 (0)	5(50)	5 (71)	10 (83)
Node metastasis, *n* (%)				
Positif	3 (33)	3 (30)	3(42)	6 (50)
Negatif	6 (67)	7 (70)	4 (57)	6 (50)
Tumor size, mean (min-max) cm	1,1 (0, 5–1, 5)	2,4 (0, 2–5, 0)	2,9 (0,1–8,0)	1,9 (0, 2–5, 0)
KI67, mean (min-max)%	8 (3–13)	39 (20–70)	44 (2–70)	50 (10–90)

We then compared by IHC the expression of CSNK1D in primary tumors with lymph nodes metastasis (N+) compared to tumors with no metastasis (N0). IHC staining was scored semi-quantitatively on the basis of intensity of the staining: 0 (non-signal); 1 (weak); 2 (moderate); and 3 (strong) staining. We found that CSNK1D expression is significantly higher in N+ primary tumors compared to N0 primary tumors (Mann Whitney test, *P* < 0.05) (Figure 2B).

To further validate these observations, we analyzed CSNK1D expression in TCGA data (Z-score retrieved via Cbioportal, [21–23]), and compared the relative expression between lymph node negative (264 samples) and lymph node positive (256 samples) breast samples. The expression of CSNK1D is significantly higher in primary tumors with metastatic lymph nodes compared to non metastatic tumors (Mann Whitney test, *P* < 0.05) (Figure 2C). These results together highlight a potential role of CSNK1D in breast cancer metastasis.

### Reduced expression of CSNK1D inhibit cancer cell proliferation, migration and invasion *in vitro*

**Figure 3:**
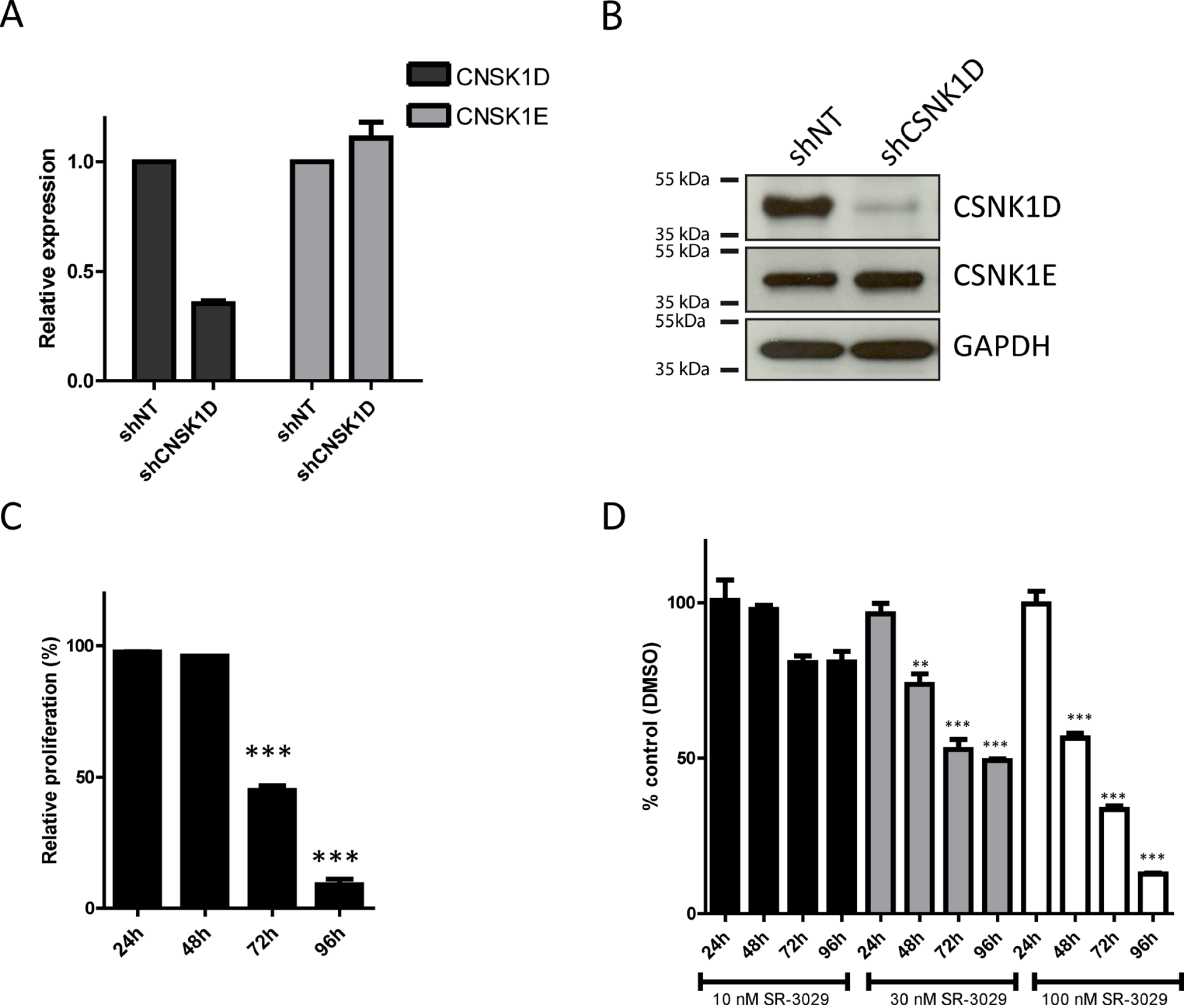
Effect of CSNK1D inactivation on proliferation of the triple negative breast cancer cell line MDA-MB-231 (**A**) Analysis by real-time PCR of CSNK1D and CSNK1E expression in the shNT or shCSNK1D cells. (**B**) Western blot analysis of CSNK1D and CSNK1E expression in shNT and shCSNK1D transduced cells. (**C**) Analysis of the proliferation of the shNT or shCSNK1D MDA-MB-231 cells. The proliferation of the shCSNK1D relative to the shNT construct is shown for a representative experiment (out of 3) with a mean of 3 replicate wells. Statistical analysis: Unpaired *t* test: ^***^=*P* < 0.001. (**D**) Effect of the inhibitor SR-3029 on the proliferation of MDA-MB-231 cells. The proliferation (relative to vehicle DMSO) in presence of the indicated concentration of SR-3029 for 24 to 96 hours is shown for a representative experiment (out of 3) with a mean of 3 replicate wells. Statistical analysis: Unpaired *t* test: ^***^=*P* < 0.001.

To study the biological significance of CSNK1D expression in cancer cells, we first examined the effect of downregulated CSNK1D expression on proliferation and motility in the highly invasive triple negative breast cancer cell line MDA-MB-231. For this, MDA-MB-231 cells were stably transduced with a control shRNA construct (shNT) or a CSNK1D targeting shRNA construct (shCSNK1D). As shown in Figure 3A and 3B, shCSNK1D transduction highly decreased CSNK1D expression at both mRNA and protein levels compared to those transduced with shNT control constructs. Keeping that CSNK1D and CSNK1E (another member of the casein kinase gene family) share more than 80% similarity, we checked the expression of CSNK1E in shCSNK1D cells. No decrease in CSNK1E expression level was observed between shCSNK1D and shNT cells (Figure 3A and 3B).

Next, we checked the effect of CSNK1D down-regulation on cell proliferation. The cell proliferation potential of the shNT or shCSNK1D MDA-MB-231 cells was measured *in vitro* by a crystal-violet assay. We found that the shCSNK1D cells had a decreased growth rate compared to the shNT cells starting at 72 hours after seeding (Figure 3C, *P* < 0.001 at 72 h and 96 hours). To further validate our results, MDA-MB-231 cells were treated with different concentration of SR-3029, a potent and selective inhibitor of CSNK1D [24]. Similarly to CSNK1D inactivation by shRNA, we observed that the specific CSNK1D inhibitor SR-3029 decreased the proliferation of MDA-MB-231 cells in a concentration dependent manner (Figure 3D: 10 nM: not significant; 30 nM: *P* < 0.01 at 48 h and *P* < 0.001 at 72 and 96 h; 100 nM: *P* < 0.001 at 48, 72 and 96 h). These results are in agreement with recently published data [20].

**Figure 4:**
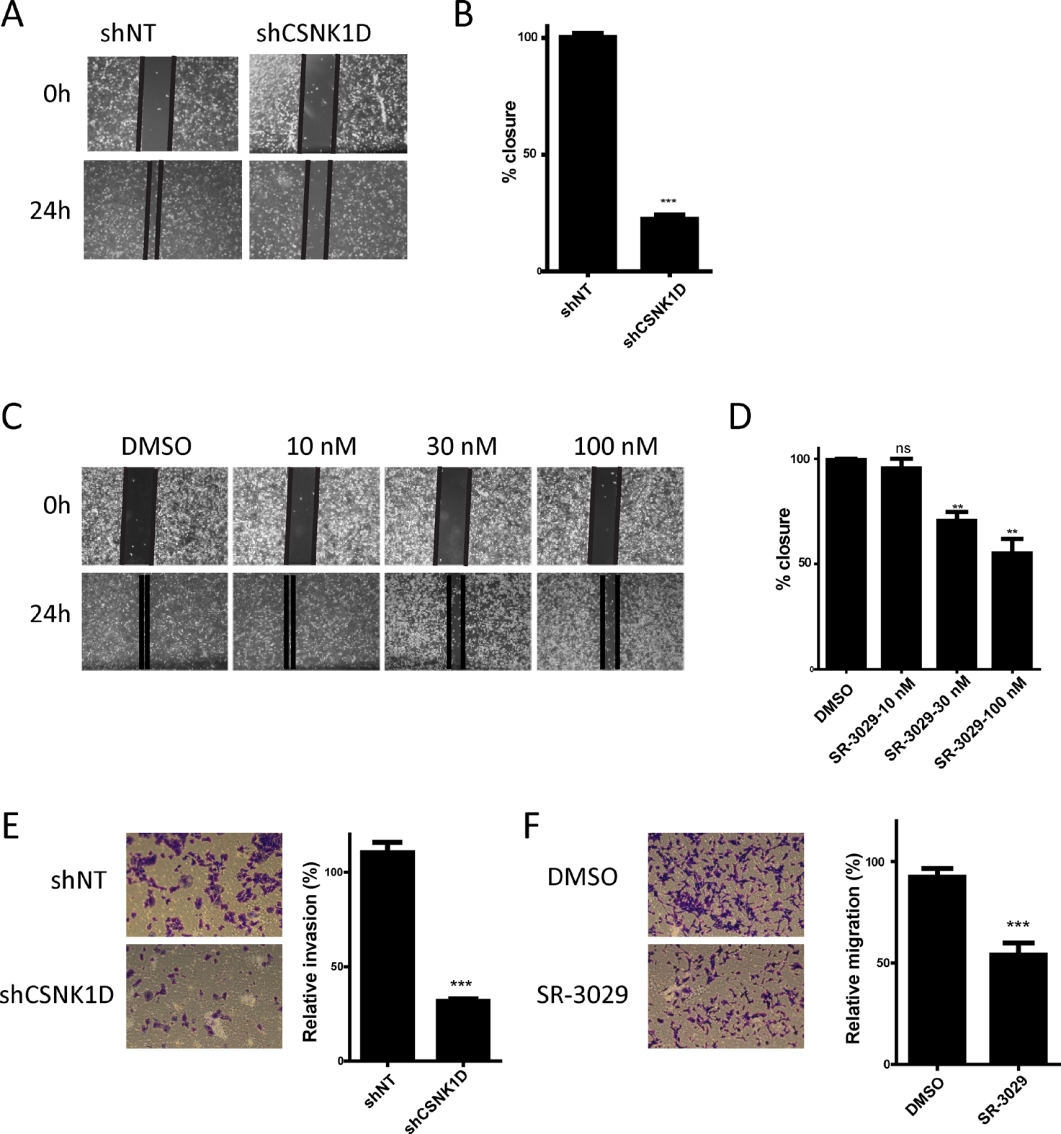
Effect of CSNK1D inactivation on the migration of the triple negative breast cancer cell line MDA-MB-231 (**A**) Representative images of the cell migration capacity of shNT and shCSNK1D analyzed using a wound healing assay over a period of 24 hours. (**B**) Quantification of the percentage of wound area closure after 24 hours in control and shCSNK1D cells. This is a representative experiment (out of 3) with a mean of 3 replicates wells. Statistical analysis: Unpaired *t* test: ^***^=*P* < 0.001. (**C**) Representative images of the cell migration capacity of MDA-MB-231 cells in presence of the indicated concentration of SR-3029 analyzed using a wound healing assay during a period of 24 hours. (**D**) Quantification of the percentage of wound area closure in presence of the indicated concentration of SR-3029 for 24 hours. This is a representative experiment (out of 3) with a mean of 3 replicates wells. Statistical analysis: Unpaired *t* test: ns not significant, ^**^=*P* < 0.01. (**E**) Comparison of the transwell migration capacity of shNT and shCSNK1D cells (representative experiment out of 3). (**F**) Analysis of the influence of 30 nM SR-3029 inhibitor of the transwell migration of MDA-MB-231 cells (representative experiment out of 3) Statistical analysis: Unpaired *t* test: ^***^=*P* < 0.001.

Keeping that motility is essential for cancer cell metastasis [25, 26], we investigated if CSNK1D contributes to the metastatic potential of breast cancer cells. For this, we examined the role of CSNK1D in MDA-MB-231 tumor cell migration and invasion. The cell migration capacity was first analyzed using a wound healing assay. For this assay, we compared the migration of shNT and shCSNK1D cells (Figure 4A and 4B) and the effect of the CSNK1D inhibitor SR-3029 on the migration of MDA-MB-231 cells (Figure 4C and 4D). Our data showed that shCSNK1D cells exhibited a slower gap closure rate than the control shNT cells (Unpaired *t* test: *P* < 0.001, Figure 4B). The cell migration was also significantly inhibited by SR-3029 in a dose dependent manner (Unpaired *t* test: *P* < 0.005 in the presence of 30 nM and 100 nM inhibitor, Figure 4D), suggesting a role for CSNK1D in cancer cell migration. Of note, no significant changes in proliferation rates were observed during the 24 hour time frame of this experiment (data not shown). The cell migration capacity was also analyzed using a transwell migration assay. As shown in Figure 4E and 4F, the migration of MDA-MB-231 is reduced in the shCSNK1D cell line or in the presence of 30 nM SR-3029 (Unpaired *t* test: *P* < 0.001).

**Figure 5:**
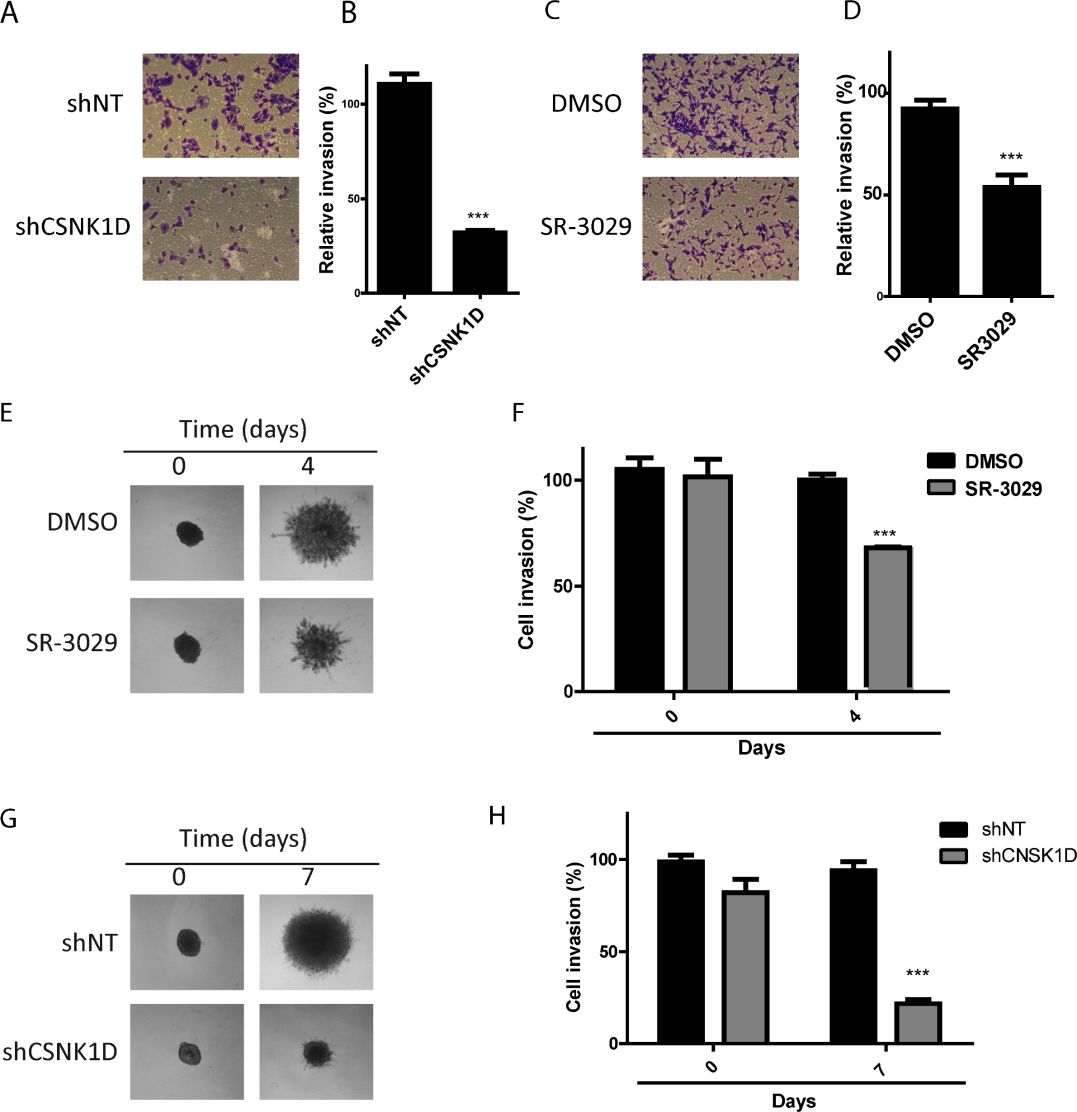
Effect of CSNK1D inactivation on matrigel invasion (**A**) Crystal violet images of shNT and shCSNK1D cells subjected to invasion assay through matrigel coated transwell. (**B**) Invasion data are calculated relative to the control shNT cells (average of 3 random microscopic images by well from 3 well replicates (1 representative experiment out of 3). Statistical analysis: Unpaired *t* test: ^***^=*P* < 0.001. (**C**) Effect of SR-3029 MDA-MB-231 cells invasion through matrigel coated transwell. (**D**) Invasion is calculated relative to vehicule (DMSO) (average of 3 random microscopic images by well from 3 well replicates (1 representative experiment out of 3). Unpaired *t* test: ^***^=*P* < 0.001. (**E**) Representative images MDA-MB-231 spheroids embedded into Matrigel and treated with 30 nM SR-3029 or vehicle (DMSO) for a period of 4 days. (**F**) Quantification using ImageJ software of a representative experiment. Unpaired *t* test: ^***^=*P* < 0.001. (**G**) Invasion across matrigel of CSNK1D depleted MDA-MB-231 spheroids (shCSNK1D or control spheroids (shNT). (**H**) Quantification using ImageJ software of a representative experiment Unpaired *t* test: ^***^=*P* < 0.001.

To further examine the effect of CSNK1D on the invasive potential of breast cancer cell line, we analyzed the invasion of MDA-MB-231 through a matrigel-coated Transwell filters. We observed that downregulation of CSNK1D expression by shRNA or inactivation of CSNK1D using the inhibitor SR-3029 significantly reduced matrigel cell invasion (Unpaired *t* test: *P* < 0.001, Figure 5A–5D).

The involvement of CSNK1D in matrigel invasion was further studied using a three-dimensional tumor spheroid invasion assay. The invasion of MDA-MB-231 spheroids in the presence or absence of 30 nM SR-3029 was monitored for 4 days. We observed a significant reduction in matrigel invasion in the presence of the CSNK1D inhibitor SR-3029 (Unpaired *t* test: *P* < 0.001, Figure 5E and 5F). The invasion of the shCSNK1D cells was also reduced compared to the invasion capacity of the control shNT cells (Unpaired *t* test: *P* < 0.001, Figure 5G and 5H).

These results together indicate that CSNK1D inactivation inhibits breast cancer cells migration and invasion *in vitro* and suggests a role of CSNK1D as a positive regulator of cancer cells motility.

### Reduced expression of CSNK1D affects the expression of invasion related genes

**Figure 6:**
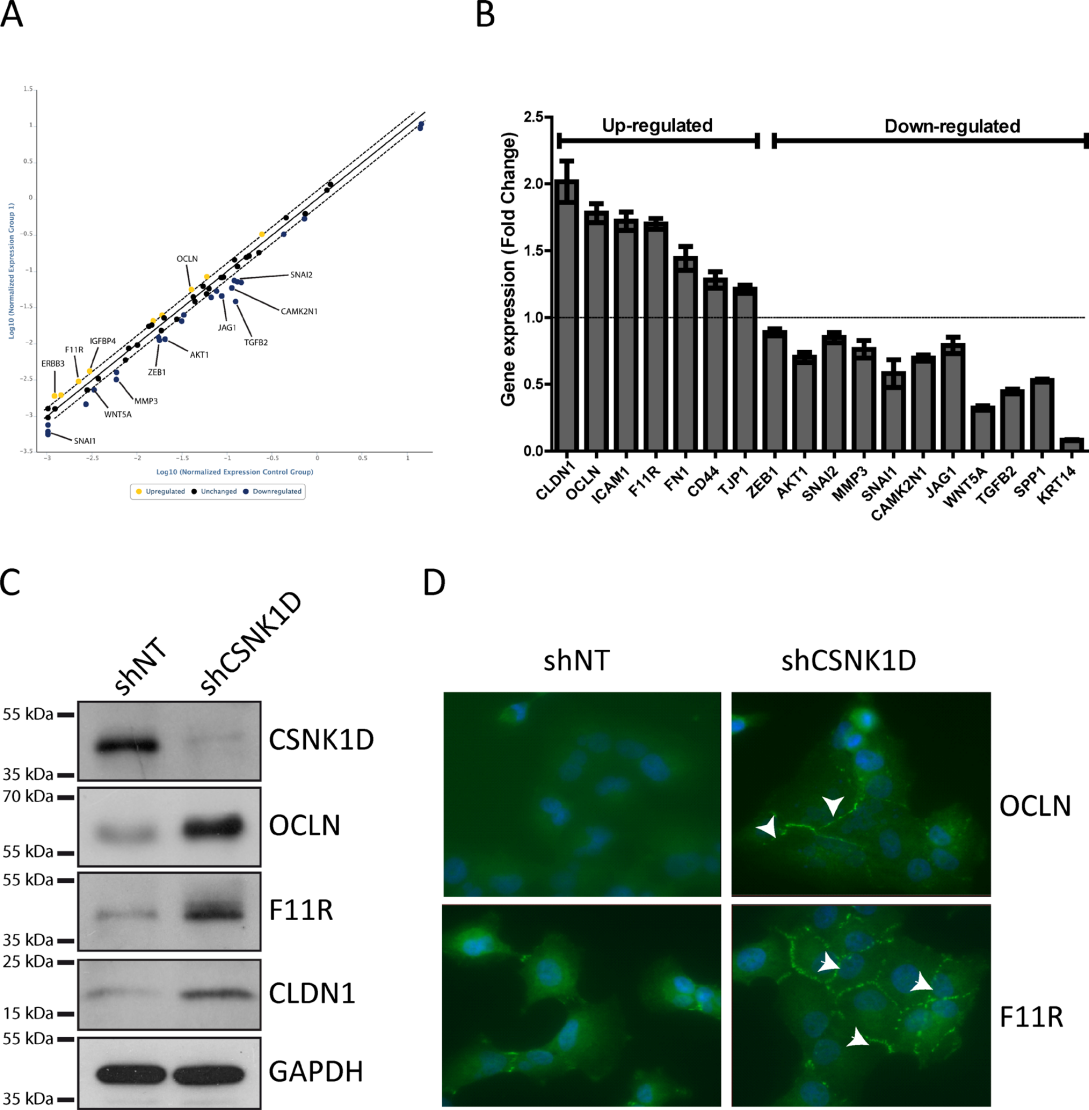
PCR array analysis and validation of genes differentially expressed between shNT and shCSNK1D cells (**A**) Scatter plot of gene expression analysis by RT^2^ Profiler PCR Array in the shNT MDA-MB-231 cells compared to shCSNK1D cells. The central line indicates unchanged gene expression. (**B**) Taqman validation of genes identified in the RT Profiler array. The histogram represents fold change of genes up-or down-regulated in shCSNK1D compared to shNT control cells. (**C**) Western blot analysis of the expression of occludin (OCLN), junction adhesion molecule A (F11R) and claudin-1 (CLDN-1) in control (shNT) and inactivated cell lines (shCSNK1D). (**D**) Detection of OCLN and F11R by immunofluorescence in shCSNK1D compared to control shNT cells. Nuclei were stained with Dapi. Arrows indicate the increased expression of tight junction proteins.

To gain mechanistic insight into the role of CSNK1D in cancer cell motility, a gene expression profiling analysis was performed between shNT and shCSNK1D transduced MDA-MB-231 cells using an RT^2^ Profiler PCR array. The PCR array contains 84 key genes implicated in epithelial–mesenchymal transition, cell growth, cell migration and motility, and also genes implicated in cytoskeleton regulation and cell adhesion. From the 84 genes analyzed in the assay, we found that the expression of 36 genes was changed in the shCSNK1D cells when compared to shNT cells. 26 of these genes were down-regulated and 10 were up-regulated (Figure 6A, Tables 2 and 3, complete list in Supplementary Table 1). Interestingly, two tight junction genes were upregulated, Occludin (OCLN) and Junctional Adhesion Molecule-A (F11R).

**Table 2:** List of downregulated genes in the PCR array, fold change >1.3

Downregulated genes			
**gene symbol**	**fold change**	**gene symbol**	**fold change**
AKT1	−1,77	RAC1	−1,36
CAMK2N1	−1,91	RGS2	−1,42
CDH2	−2,02	GEMIN2	−1,44
COL3A1	−1,65	SNAI2	−1,82
ESR1	−1,39	SPP1	−4,54
FOXC2	−2,43	TFPI2	−2,04
GNG11	−1,84	TGFB2	−3,23
JAG1	−1,91	TSPAN13	−1,47
KRT14	−3,66	VIM	−1,48
MMP3	−1,83	VPS13A	−1,52
MST1R	−1,5	WNT5A	−2,54
PLEK2	−1,52	WNT5B	−1,44
DESI1	−1,6	ZEB1	−1,58

**Table 3:** List of upregulated genes in the PCR array, fold change >1.3

Up-regulated genes	
**gene symbol**	**fold change**
COL5A2	1,37
ERBB3	1,58
F11R	1,35
FGFBP1	1,75
IGFBP4	1,41
ITGAV	1,37
KRT7	1,42
NODAL	1,46
OCLN	1,4
TGFB3	1,44

We then performed independent RT-qPCR to confirm the change in expression of several genes of the RT profiler array. Among the genes tested, we confirmed a positive correlation between the expression of CSNK1D and the expression of TGF-β2, WNT5A, SNAIL1, AKT1 and CAMK2N (Figure 6B). The expression of cytokeratin 14 was also downregulated in shCSNK1D cells compared to shNT (Figure 6B).

Keeping that the tight junction is a key element in tumor progression and metastasis, we focused on the expression of tight junction genes including genes not present in the array (Figure 6B). The RT-qPCR data showed that CSNK1D inactivation correlates with an increase in the expression of the tight junction genes Claudin-1 (CLDN1), OCLN and F11R. We then further examined whether the increase in gene expression is accompanied by an increase of protein level. Similar to RT-qPCR results, CSNK1D knock-down increased the protein level of tight junction proteins as confirmed by immunoblotting and immunofluorescence (Figure 6C and 6D).

All the above data together indicate that CSNK1D regulates tight junction proteins expression at the mRNA and protein level in MDA-MB-231 cells and thereby might be implicated in the invasiveness of breast cancer cells.

### Reduced expression of CSNK1D impairs metastasis *in vivo*

**Figure 7:**
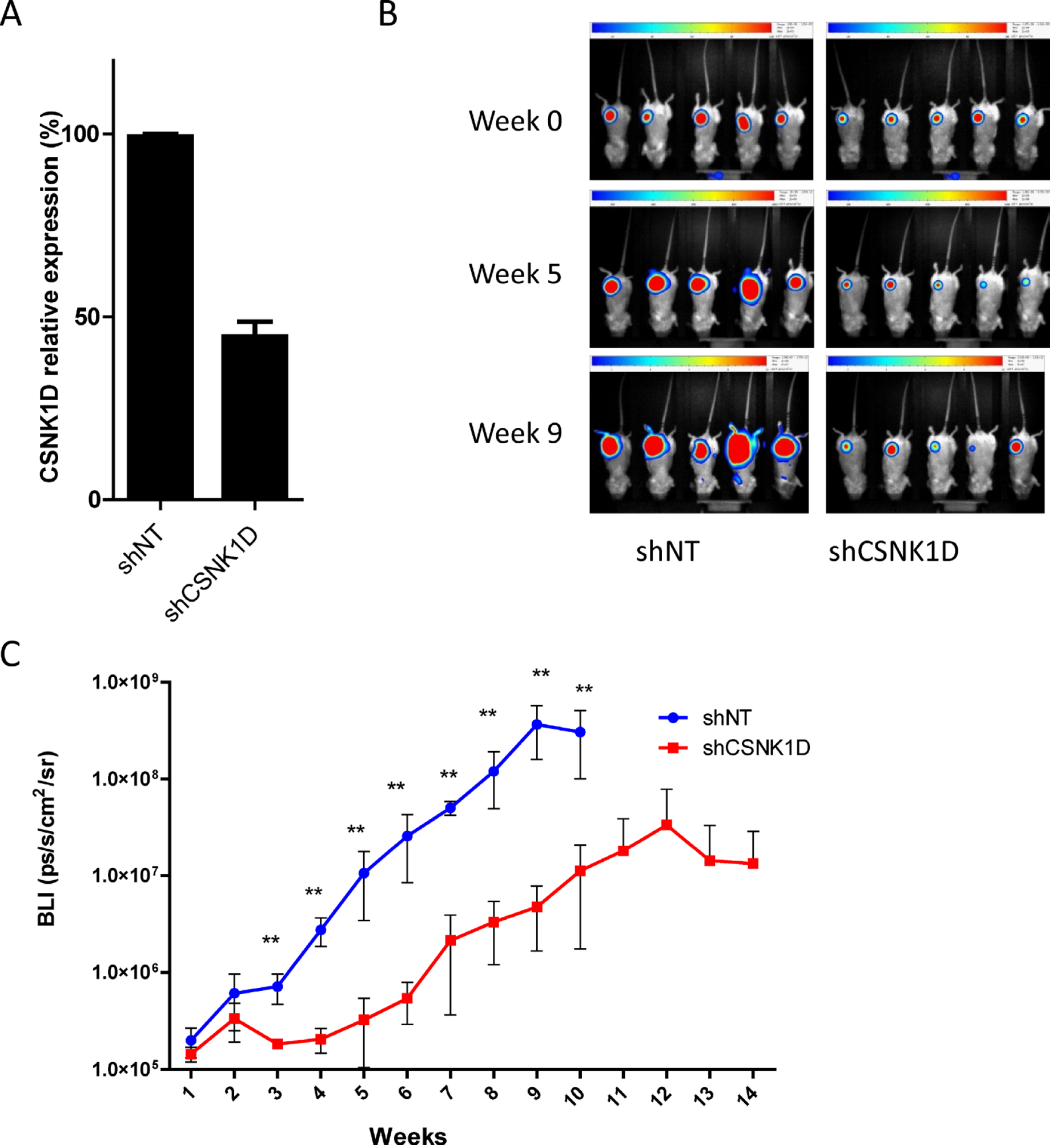
*In vivo* tumor growth monitoring after mammary fat pad injection of MDA-MB-231 cells transduced with shNT or shCSNK1D cells in immunodeficient mice (**A**) RT-qPCR analysis of CSNK1D knock-down in MDA-MB-231 expressing the firefly luciferase. (**B**) Representative BLI images of tumor xenografts in Scid Beige mice, at day 0 post injection, week 5 and week 9. (**C**) BLI signal intensities analysis of tumor development after implantation into the axillary mammary fat pad of Scid Beige mice (Mann–Whitney test: ^**^p < 0.01).

Since cells with altered expression of CSNK1D also have a reduced migration and invasion, we tested whether this could also affect the metastatic capacity of breast cancer cells in addition to the effect on primary tumor growth. MDA-MB-231 cells are highly metastatic and are used as a model system to study metastasis of breast cancer. We investigated the effect of CSNK1D on metastasis by injecting MDA-MB-231 cells expressing the firefly luciferase and transduced with control shNT (*n* = 5) or shCSNK1D (*n* = 5) into the mammary fat pad of immunodeficient SCID Beige mice. A reduction of CSNK1D expression was confirmed by RT-qPCR (Figure 7A). The tumor growth was monitored *in vivo* by bioluminescence imaging (BLI) until signal stopped to increase. As illustrated in Figure 7B and 7C, shCSNK1D tumor development is slower than the development of shNT tumors. The difference in BLI intensities was significant starting from week 2 after injection (Figure 7C) (*p <* 0.01). In keeping with previous observations [20], these results confirmed the effect of CSNK1D inhibition on tumor growth *in vivo*.

**Figure 8:**
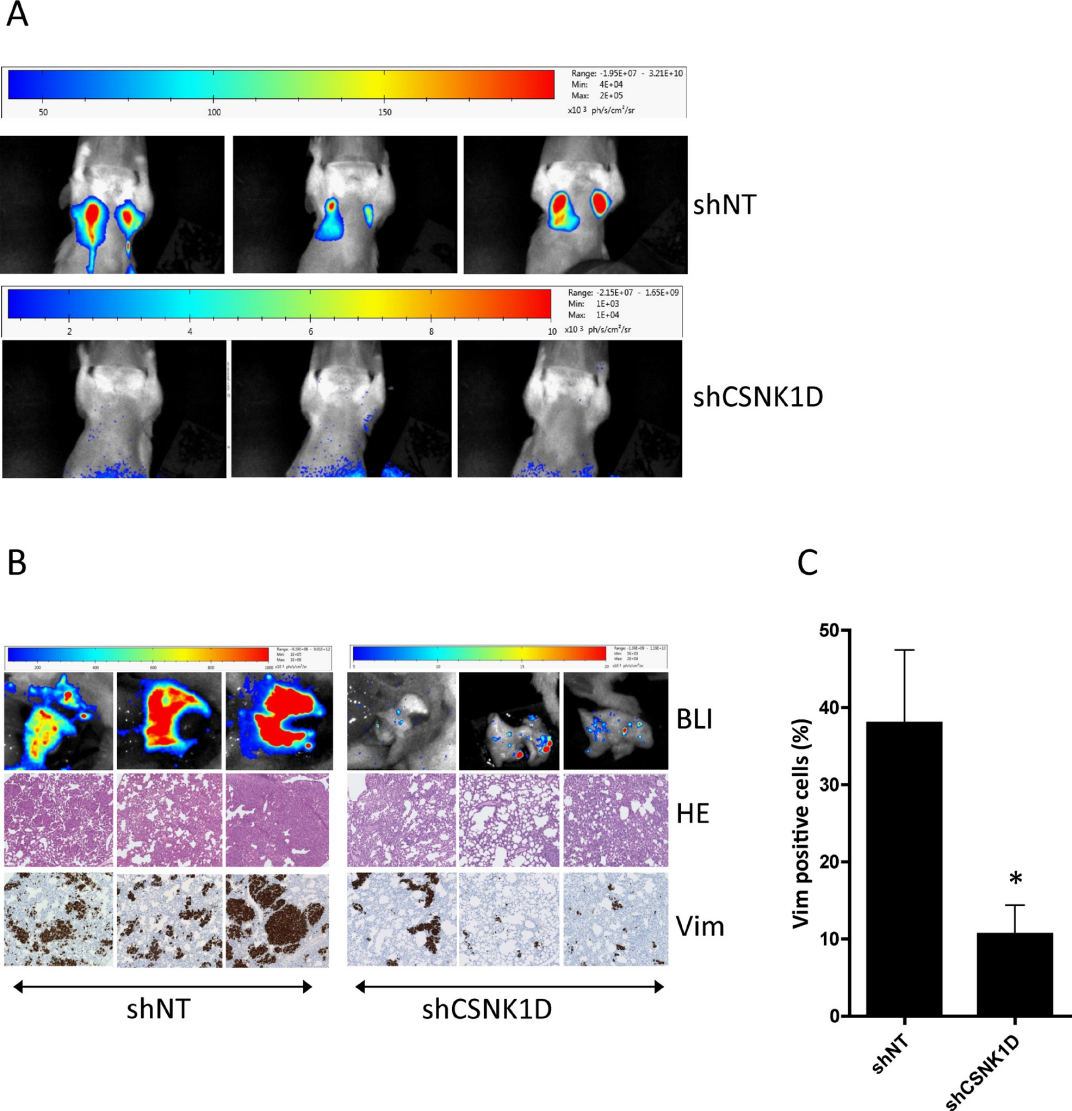
Spontaneous metastasis from primary fat pads tumors to lung (**A**) *In vivo* BLI analysis 9 weeks after mammary fat pad injection of MDA-MB-231 cells transduced with shNT or shCSNK1D cells. (**B**) *Ex vivo* metastasis detection by BLI at week 9 (shNT) or week 13 (shCSNK1D). Metastasis to lung was confirmed by hematoxylin-eosin staining and vimentin IHC. (**C**) Percentage of vimentin positive cells in the lung of control and shCSNK1D mice. Data are mean ± SEM. Statistical analysis: Unpaired *t* test: ^*^= *p <* 0.05.

To check the effect of CSNK1D on metastasis, *in vivo* BLI was performed at week 9 (Figure 8A). In the shNT group at week 9, high intensities metastasis were detected in the lungs for 4/5 mice. No metastasis was detected at that time point in the shCSNK1D group. *Ex vivo* BLI evaluations were also performed on mice organs at the sacrifice time point (week 9 for shNT group and week 13 for shCSNK1D group) before histological evaluations. *Ex vivo* BLI showed a high content of metastasis in the lungs of 4/5 mice of the shNT group (Figure 8B). On the contrary, in the shCSNK1D group, few positives nodules were found in 4/5 lungs (Figure 8B). In addition, vimentin immunohistochemistry on tissues sections confirmed that 4/5 lungs had numerous nodules in shNT group, while lung infiltration was significantly lower in the shCSNK1D group Figure 8B, 8C). The quantification of lung colonization was assayed by calculating the total area of lung metastasis lesion, as detected by vimentin IHC, normalized to the total area of the lungs (*p <* 0.05, Figure 8C). Together, these findings highlight an important role for CSNK1D in metastasis of breast cancer and provide evidences that CSNK1D inactivation specifically prevents metastasis of cancer cells.

## DISCUSSION

Tumor metastasis in breast cancer indicates aggressiveness and poor prognosis. This study provides evidences that the kinase CSNK1D is implicated in breast cancer cell growth, migration, invasion and metastasis. We show that CSNK1D knock-down by shRNA in MDA-MB-231 breast cancer cells significantly inhibit cell migration and invasion. In addition, the reduced expression of CSNK1D increases the expression of the tight junction components at the mRNA and protein level in the highly metastatic triple negative cell line MDA-MB-231. Finally, we show that CSNK1D knock-down significantly reduces tumor growth and lung metastasis of human breast cancer cells in xenograft model.

While previous studies have determined that CSNK1D plays a role in breast cancer cell proliferation, its role in cell migration, invasion and metastasis has not been investigated. It was reported that CSNK1D overexpression correlates with lymph node positive breast carcinoma and that impaired CSNK1D affects mammary tumorigenesis *in vivo* [27, 28]. Our results confirmed that the CSNK1D protein is highly expressed in primary breast tumors and matched metastatic lymph nodes and further support a potential role for CSNK1D in breast cancer metastasis.

We showed that the regulation of CSNK1D expression is important for the maintenance of normal tight junction. Accumulating evidences indicate that the disruption of the tight junction structure is associated with cancer progression and metastasis [29]. The tight junctions are composed of several proteins comprising the transmembrane proteins occludin and claudins in association with junctional adhesion molecules which interact with proteins such as zonula occludens protein and the actin cytoskeleton [29]. During epithelial to mesenchymal transition there is a decrease in claudin and occludin expression, and a diffusion of zonula occludens 1 (ZO1; also known as TJP1) from cell–cell contact. These modifications in the expression of several tight junction proteins have been reported to be associated with progression and metastasis in breast and prostate cancer [30, 31]. Loss of Claudin-1, a member of the tight junction, is associated with cancer invasion and the acquisition of the metastatic phenotype in breast cancer [32]. Similarly, loss of occluding expression is associated with poor prognosis and metastasis [31, 33, 34]. The junctional adhesion molecule A (JAM-A), a tight junction protein, is also a key negative regulator of cell migration and invasion in breast cancer [35–37]. We previously reported that CSNK1D is frequently amplified in triple negative breast cancer [4]. Ours results indicate that CSNK1D inactivation specifically prevents metastasis of cancer cells and correlates with increased tight junction proteins expression. The exact mechanism behind the effect of CSNK1D on metastasis remains to be evaluated. However, it is tempting to hypothesize that this is at least partially mediated by the regulation of tight junction protein expression.

The deregulation of CSNK1D expression or function has been observed in cancer, but also in several other pathologies like Alzheimer's disease or familial advanced sleep phase syndrome [38, 39]. Given the importance of CSNK1D in these pathologies, numerous efforts have been investigated into the development and characterization of CSNK1D specific inhibitors [8] [40, 24]. These inhibitors could be of interest to develop targeted cancer therapies.

In conclusion, our results suggest that CSNK1D might influence the progression of breast cancer and metastasis via the regulation of tight junction proteins expression and might represent a potential therapeutic target in breast cancer.

## MATERIALS AND METHODS

### Cell culture

The highly metastatic triple negative cell line MDA-MB-231 was purchased from ATCC and maintained in Dulbecco's Modified Eagles Medium (Invitrogen) supplemented with 10% fetal bovine serum. Cells were maintained in an incubator with humidified air (5% CO2) at 37°C.

### Reagents and antibodies

Crystal violet was purchased from Sigma (#3886). The SR-3029 inhibitor was obtained from Glixx Laboratories Inc. Antibodies for CSNK1D (#sc-55553), CSNK1E (#sc-25423), Occludin (#sc-133256), JAM-A (#sc-53623) and GAPDH (#sc-365062) were from Santa-Cruz. Claudin-1 antibody (#13255), horseradish-peroxidase-conjugated anti-rabbit (#7074) and anti-mouse-IgG (#7076) were from Cell signaling.

### knock-down of CSNK1D using short hairpin RNAs

Lentiviral vectors and transduced cells were generated in the GIGA Viral Vectors platform (University of Liège). The pLKO-1 plasmids with specific shRNAs were obtained from Sigma Aldrich. MDA-MB-231 cells were stably transduced with a shRNA construct targeting CSNK1D (TRCN0000194725, shCSNK1D) and selected with puromycin (0.5 mg/ml). A non targeting construct was used as control (Sigma-Aldrich, SHC016, shNT).

### Cell proliferation assay

MDA-MB-231 cells transduced with the control shNT or the shCSNK1D targeting CSNK1D, or treated with SR-3029, were seeded at a density of 5 × 10^4^ cells/well in 12-well plate and incubated at 37°C with 5% CO2. After 24, 48, 72 and 96 hours of culture, the cells were washed in PBS, fixed in 4% formaldehyde for 15 minutes and stained with 0.1% crystal violet for 20 minutes. Cells were washed with water and staining was extracted with 10% acetic acid for 20 minutes and absorbance was measured at 590 nm. The experiments were performed in triplicates.

### Cell migration assay

IBIDI culture inserts (IBIDI GmbH) were placed into 24 well plate. An equal number of shNT and shCSNK1D cells (70 μl; 4 × 10^5^ cells/ml) were added into the culture inserts and incubated at 37°C/5% CO_2_. After overnight incubation, the inserts were removed; the wells were washed and filled with complete growth medium. Pictures were taken immediately and again 24 hours after the creation of the wound, using a Leica inverted microscope. The experiments were performed in triplicate and wound closure was analyzed using the TScratch software [41].

### Transwell cell invasion and migration assay

The cells were starved in 0.5% serum- DMEM during 8 hours. For migration assay, a total of 5 × 10^4^ cells were suspended in 500 μl of serum-free DMEM medium and seeded into the upper chamber of a 8 μM pore size insert (24-well insert; BD Biocoat control inserts). Then, 750 μL of DMEM containing 10% FBS with or without inhibitors was added to the lower compartment. After incubation at 37°C for 24 h, migrated cells were washed with PBS, fixed in 4% PFA and stained for 30 min in crystal violet solution (0.1% crystal violet). Cells that did not migrate to the lower compartment were removed with a cotton swab. Each insert was photographed in 3 random fields at a magnification of 40×.

For invasion assay, a total of 1.5 × 10^5^ cells were suspended in 500 μl of serum-free DMEM medium and seeded into the upper chamber of a 8 μM pore size transwell inserts pre-coated with Matrigel (24 well inserts, Corning BioCoat Growth Factor Reduced Matrigel Invasion Chamber). After incubation at 37°C for 24 h the invading cells were analyzed as described above.

### 3-D spheroid culture and invasion assay

The 3-D invasion assay was performed using the Cultrex 3D Culture BME Cell Invasion Assay, 96 wel (RD Systems) according to manufacturer's instruction.

Briefly, cells were plated at a density of 5000 cells per well in the spheroid formation medium and grown for 3 days at 37°C with 5% CO_2_. Spheres were starved in 0.5% serum in DMEM for 4 hours. Then the matrigel invasion matrix was added to each well and the cells were incubated at 37°C for 1 hour. Fresh DMEM medium containing 20% serum with or without inhibitors was then added and the plates were incubated at 37°C to evaluate the invasion. Images were captured at different time interval. Invasive protrusions were quantified using ImageJ software (NIH) according to the manufacturer instructions.

### RT^2^ Profiler array analysis

The mRNA expression of 84 genes in MDA-MB-231 cells transfected with shNT or shCSNK1D was analyzed using the Human Epithelial to Mesenchymal Transition RT2 Profiler PCR Arrays (Qiagen). 500 ng of total RNA was reverse transcribed using the RT2 First Strand Kit (Qiagen) according to the manufacturer's instructions. Quantitative RT–PCR was carried out on a LightCycler 480 (Roche Diagnostics) using RT2 Real-Time SYBR Green qPCR Master Mix (SABiosciences, Qiagen). Results analyses were performed using the web-based PCR Array Data Analysis Software tool. Geometric mean of the housekeeping genes ACTB, GAPDH and RPLP0 was used for normalization and fold change was used to analyze differences in gene expression.

### RNA isolation, cDNA synthesis and individual Taqman qRT-PCR

Total RNA was isolated from cells using the Maxwell 16 Total RNA Purification Kit (Promega) according to the manufacturer's protocol. One microgram of total RNA was reverse transcribed into cDNA using the “SuperScript III First-Strand Synthesis superMix (Invitrogen) according to the manufacturer's recommendations. The qRT-PCR reactions were performed using 20 ng of cDNA and the ABsolute Blue qPCR Mix, low ROX (Thermo Scientific) and run on a Lightcycler 480 (RocheDiagnostics). Pre-designed Taqman assays were ordered from IDT. *RPL37A* was chosen as housekeeping gene. Transcript levels were expressed as 2^−ΔΔCt^.

### Protein assays

Proteins were extracted in RIPA buffer (Amresco) supplemented with phosphatases and protease inhibitors (Cell Signaling), and Western blotting was performed using standard protocol. Equal amounts of total cell protein (15 *μ*g per lane) were electrophoresed on SDS–polyacrylamide gradient gels (4–15% Mini-protean TGX TM gel, Bio-Rad laboratories) and transferred to nitrocellulose membranes (Amersham). Detection was performed using the indicated antibodies.

The primary antibodies were detected with horseradish-peroxidase-conjugated anti-rabbit or anti-mouse-IgG secondary antibodies followed by measurement of chemoluminescence (Lumi-LightPLUS, Roche).

### Immunofluorescence

Cells were fixed in 4% PFA at RT for 10 minutes, washed with PBS and subsequently permeabilized with 0.3% Triton X-100 in PBS for 5 minutes. Cells were blocked with 5% normal goat serum in PBS for 1 hour and incubated with primary antibody overnight at 4°C. Slides were washed and incubated with anti-rabbit or anti-mouse (Alexa Fluor 488 Conjugate, Cell Signaling, 1:250) for 1 hour at room temperature. After washing, the cells were mounted with Prolong gold antifade medium with DAPI (Cell Signaling).

### *In vivo* metastasis assays

MDA-MB-231 cells, which were engineered to stably express firefly luciferase, were transduced with the shCSNK1D construct or the control shNT construct (GIGA Viral Vectors platform, Liège).

The cells were injected (5 × 10^5^ cells) subcutaneously into the mammary fat pad of immunodeficient Scid Beige mice (*N* = 5 per condition). The tumor growth was monitored weekly by bioluminescence imaging (BLI) in a PhotonImager RT (Biospace Lab), following subcutaneous injection of 150 mg/kg of D-Luciferin (Promega). Monitoring was ended once the signal stopped to increase (week 10 for the control condition and week 14 for the shCSNK1D condition). At week 9, each mouse was imaged by BLI individually using the maximal zoom for metastasis detection. To avoid signal overlapping and to maximize sensitivity, the primary tumor was hided with a cap. Mice of both groups were then sacrificed (at week 10 and 14 respectively), opened and directly imaged *ex vivo* by BLI. The tumor and different organs (nodes, lung, ovary, spleen and liver) were dissected and fixed in 4% paraformaldehyde before paraffin embedding.

Animal experiments were performed under approval of the ethics committee of the Center for Microscopy and Molecular Imaging (CMMI protocol number 2011–08; LA1500589).

### Immunohistochemitry

Antigen retrieval was performed in a PT-link pre-treatment module (DAKO) on 5 μM FFPE sections. After endogenous peroxidase blocking, sections were incubated overnight at 4°C with a CSNK1D primary antibody directed against the C-terminal region (1:50 dilution, sc-55553, Santa-Cruz). After three washes with TBS–Tween, sections were incubated with HRP conjugated anti-mouse polymer (Envision, DAKO) for 30 minutes at room temperature and immunoreactivity was revealed using 3′3′-diaminobenzidine (DAB).

Detection of Vimentin on mouse lung was performed on an Omnis automate with ready to use antibody clone V9 (DAKO). The number of vimentin positive cells was determined using the HALO image analysis program (Indica Labs).

### RNA *In situ* hybridization (ISH) assay

ISH for *CSNK1D* mRNA was performed using the RNAscope 2.0 FFPE assay kit (Advanced cell diagnostics) according to the manufacturer's instructions. Sections were deparaffinized with xylene and dehydrated with ethanol 100%. Endogenous peroxidase activity was blocked with pre-treatment 1 solution and protease digestion was performed using pre-treatment 2 solution. These steps were followed by hybridization with probes targeting *CSNK1D* mRNAs. mRNA coding for the housekeeping RNA polymerase II (POLR2A) was used as a positive control and the bacterial gene *DapB* as a negative control.

### Tumor samples

Formalin-fixed paraffin-embedded (FFPE) tumors were selected from the biobank of the Institute of Pathology and Genetics (IPG, Gosselies). CSNK1D staining was performed on 9 LumA, 10 LumB, 7 HER2 and 12 TN breast tumors. All experiments involving human tissues were conducted with the permission of the ethics committee of the “Grand Hôpital de Charleroi” (GHdC). Patients’ characteristics are summarized in Table 1.

## SUPPLEMENTARY MATERIALS TABLE





## Abbreviations

ACTB: actin B
BC: Breast cancer
BLI: bioluminescence imaging
CLN1: claudin 1
CSNK1A: casein kinase 1 alpha
CSNK1D: casein kinase 1 delta
CSNK1E: casein kinase 1 epsilon
DAB: 3, 3-diaminobenzidine
F11R: F11 Receptor, Junctional adhesion molecule-A
FFPE: Formalin-fixed paraffin-embedded
GAPDH: Glyceraldehyde-3-Phosphate Dehydrogenase
HE: Hematoxylin and eosin
HER2: Human epidermal growth factor receptor 2
HRP: Horseradish peroxidase
IHC: Immunohistochemistry
ISH: *In situ* hybridization
JAM-A: Junctional adhesion molecule-A
LumA: Luminal A
LumB: Luminal B
OCLN: Occludin
PBS: Phosphate-buffered saline
shRNA: short hairpin RNA
TBS: Tris-buffered saline
TJP1: tight junction protein 1
TN: triple negative
TNBC: triple negative breast cancer
VIM: vimentin

## References

[1] Denkert C, Liedtke C, Tutt A, von Minckwitz G (2017). Molecular alterations in triple-negative breast cancer-the road to new treatment strategies. Lancet.

[2] Yam C, Mani SA, Moulder SL (2017). Targeting the Molecular Subtypes of Triple Negative Breast Cancer: Understanding the Diversity to Progress the Field. Oncologist.

[3] Anders CK, Carey LA (2009). Biology, metastatic patterns, and treatment of patients with triple-negative breast cancer. Clin Breast Cancer.

[4] Toffoli S, Bar I, Abdel-Sater F, Delrée P, Hilbert P, Cavallin F, Moreau F, Van Criekinge W, Lacroix-Triki M, Campone M, Martin AL, Roché H, Machiels JP (2014). Identification by array comparative genomic hybridization of a new amplicon on chromosome 17q highly recurrent in BRCA1 mutated triple negative breast cancer. Breast Cancer Res.

[5] Cheong JK, Virshup DM (2011). Casein kinase 1: complexity in the family. Int J Biochem Cell Biol.

[6] Knippschild U, Wolff S, Giamas G, Brockschmidt C, Wittau M, Würl PU, Eismann T, Stöter M (2005). The role of the casein kinase 1 (CK1) family in different signaling pathways linked to cancer development. Onkologie.

[7] Schittek B, Sinnberg T (2014). Biological functions of casein kinase 1 isoforms and putative roles in tumorigenesis. Mol Cancer.

[8] Knippschild U, Krüger M, Richter J, Xu P, García-Reyes B, Peifer C, Halekotte J, Bakulev V, Bischof J (2014). The CK1 family: contribution to cellular stress response and its role in carcinogenesis. Front Oncol.

[9] Sinnberg T, Wang J, Sauer B, Schittek B (2016). Casein kinase 1α has a non-redundant and dominant role within the CK1 family in melanoma progression. BMC Cancer.

[10] Sinnberg T, Menzel M, Kaesler S, Biedermann T, Sauer B, Nahnsen S, Schwarz M, Garbe C, Schittek B (2010). Suppression of casein kinase 1α in melanoma cells induces a switch in β-catenin signaling to promote metastasis. Cancer Res.

[11] Manni S, Carrino M, Piazza F (2017). Role of protein kinases CK1α and CK2 in multiple myeloma: regulation of pivotal survival and stress-managing pathways. J Hematol Oncol.

[12] Bidère N, Ngo VN, Lee J, Collins C, Zheng L, Wan F, Davis RE, Lenz G, Anderson DE, Arnoult D, Vazquez A, Sakai K, Zhang J (2009). Casein kinase 1alpha governs antigen-receptor-induced NF-kappaB activation and human lymphoma cell survival. Nature.

[13] Lopez-Guerra JL, Verdugo-Sivianes EM, Otero-Albiol D, Vieites B, Ortiz-Gordillo MJ, De León JM, Praena-Fernandez JM, Marin JJ, Carnero A (2015). High casein kinase 1 epsilon levels are correlated with better prognosis in subsets of patients with breast cancer. Oncotarget.

[14] Lin SH, Lin YM, Yeh CM, Chen CJ, Chen MW, Hung HF, Yeh KT, Yang SF (2014). Casein kinase 1 epsilon expression predicts poorer prognosis in low T-stage oral cancer patients. Int J Mol Sci.

[15] Lin SH, Yeh CM, Hsieh MJ, Lin YM, Chen MW, Chen CJ, Lin CY, Hung HF, Yeh KT, Yang SF (2016). Low cytoplasmic casein kinase 1 epsilon expression predicts poor prognosis in patients with hepatocellular carcinoma. Tumour Biol.

[16] Foldynová-Trantírková S, Sekyrová P, Tmejová K, Brumovská E, Bernatík O, Blankenfeldt W, Krejcí P, Kozubík A, Dolezal T, Trantírek L, Bryja V (2010). Breast cancer-specific mutations in CK1epsilon inhibit Wnt/beta-catenin and activate the Wnt/Rac1/JNK and NFAT pathways to decrease cell adhesion and promote cell migration. Breast Cancer Res.

[17] Brockschmidt C, Hirner H, Huber N, Eismann T, Hillenbrand A, Giamas G, Radunsky B, Ammerpohl O, Bohm B, Henne-Bruns D, Kalthoff H, Leithäuser F, Trauzold A, Knippschild U (2008). Anti-apoptotic and growth-stimulatory functions of CK1 delta and epsilon in ductal adenocarcinoma of the pancreas are inhibited by IC261 in vitro and in vivo. Gut.

[18] Qin YN, He DM, Zhang ZY, Yu XH (2017). Aberrant expression of casein kinase 1 delta (CK1 delta) in cervical squamous cell carcinoma. Int J Clin Exp Pathol.

[19] Richter J, Rudeck S, Kretz AL, Kramer K, Just S, Henne-Bruns D, Hillenbrand A, Leithäuser F, Lemke J, Knippschild U (2016). Decreased CK1δ expression predicts prolonged survival in colorectal cancer patients. Tumour Biol.

[20] Rosenberg LH, Lafitte M, Quereda V, Grant W, Chen W, Bibian M, Noguchi Y, Fallahi M, Yang C, Chang JC, Roush WR, Cleveland JL, Duckett DR (2015). Therapeutic targeting of casein kinase 1δ in breast cancer. Sci Transl Med.

[21] Gao J, Aksoy BA, Dogrusoz U, Dresdner G, Gross B, Sumer SO, Sun Y, Jacobsen A, Sinha R, Larsson E, Cerami E, Sander C, Schultz N (2013). Integrative analysis of complex cancer genomics and clinical profiles using the cBioPortal. Sci Signal.

[22] Cerami E, Gao J, Dogrusoz U, Gross BE, Sumer SO, Aksoy BA, Jacobsen A, Byrne CJ, Heuer ML, Larsson E, Antipin Y, Reva B, Goldberg AP (2012). The cBio cancer genomics portal: an open platform for exploring multidimensional cancer genomics data. Cancer Discov.

[23] Cancer Genome Atlas Network (2012). Comprehensive molecular portraits of human breast tumours. Nature.

[24] Bibian M, Rahaim RJ, Choi JY, Noguchi Y, Schürer S, Chen W, Nakanishi S, Licht K, Rosenberg LH, Li L, Feng Y, Cameron MD, Duckett DR (2013). Development of highly selective casein kinase 1δ/1ε (CK1δ/ε) inhibitors with potent antiproliferative properties. Bioorg Med Chem Lett.

[25] Scully OJ, Bay BH, Yip G, Yu Y (2012). Breast cancer metastasis. Cancer Genomics Proteomics.

[26] Pandya P, Orgaz JL, Sanz-Moreno V (2017). Modes of invasion during tumour dissemination. Mol Oncol.

[27] Abba MC, Sun H, Hawkins KA, Drake JA, Hu Y, Nunez MI, Gaddis S, Shi T, Horvath S, Sahin A, Aldaz CM (2007). Breast cancer molecular signatures as determined by SAGE: correlation with lymph node status. Mol Cancer Res.

[28] Hirner H, Günes C, Bischof J, Wolff S, Grothey A, Kühl M, Oswald F, Wegwitz F, Bösl MR, Trauzold A, Henne-Bruns D, Peifer C, Leithäuser F (2012). Impaired CK1 delta activity attenuates SV40-induced cellular transformation in vitro and mouse mammary carcinogenesis in vivo. PLoS One.

[29] Knights AJ, Funnell AP, Crossley M, Pearson RC (2012). Holding tight: cell junctions and cancer spread. Trends Cancer Res.

[30] Martin TA, Mason MD, Jiang WG (2011). Tight junctions in cancer metastasis. Front Biosci.

[31] Martin TA, Mansel RE, Jiang WG (2010). Loss of occludin leads to the progression of human breast cancer. Int J Mol Med.

[32] Swisshelm K, Macek R, Kubbies M (2005). Role of claudins in tumorigenesis. Adv Drug Deliv Rev.

[33] Martin TA, Watkins G, Mansel RE, Jiang WG (2004). Loss of tight junction plaque molecules in breast cancer tissues is associated with a poor prognosis in patients with breast cancer. Eur J Cancer.

[34] Martin TA, Jordan N, Davies EL, Jiang WG (2016). Metastasis to bone in human cancer is associated with loss of occludin expression. Anticancer Res.

[35] Naik MU, Naik TU, Suckow AT, Duncan MK, Naik UP (2008). Attenuation of junctional adhesion molecule-A is a contributing factor for breast cancer cell invasion. Cancer Res.

[36] Mathe A, Scott RJ, Avery-Kiejda KA (2015). miRNAs and other epigenetic changes as biomarkers in triple negative breast cancer. Int J Mol Sci.

[37] McSherry EA, McGee SF, Jirstrom K, Doyle EM, Brennan DJ, Landberg G, Dervan PA, Hopkins AM, Gallagher WM (2009). JAM-A expression positively correlates with poor prognosis in breast cancer patients. Int J Cancer.

[38] Li G, Yin H, Kuret J (2004). Casein kinase 1 delta phosphorylates tau and disrupts its binding to microtubules. J Biol Chem.

[39] Brennan K, Bates EA, Shapiro RE, Zyuzin J, Hallows WC, Huang Y, Lee HY, Jones CR, Fu YH, Charles AC (2013). Casein kinase iδ mutations in familial migraine and advanced sleep phase. Sci Transl Med.

[40] Halekotte J, Witt L, Ianes C, Krüger M, Bührmann M, Rauh D, Pichlo C, Brunstein E, Luxenburger A, Baumann U, Knippschild U, Bischof J, Peifer C (2017). Optimized 4,5-Diarylimidazoles as Potent/Selective Inhibitors of Protein Kinase CK1δ and Their Structural Relation to p38α MAPK. Molecules.

[41] Gebäck T, Schulz MM, Koumoutsakos P, Detmar M (2009). TScratch: a novel and simple software tool for automated analysis of monolayer wound healing assays. Biotechniques.

