# Supplementary material for: Silencing of casein kinase 1 delta reduces migration and metastasis of triple negative breast cancer cells
Source: Oncotarget. 2018 Jul 20;9(56):30821–36. doi: 10.18632/oncotarget.25738 (PMC6089398; doi:10.18632/oncotarget.25738)
Supplement: Supplementary file 1 [file oncotarget-09-30821-s001.pdf]

Silencing of casein kinase 1 delta reduces migration and metastasis of triple negative breast cancer cells

SUPPLEMENTARY MATERIALS

Supplementary Table 1 : RT2 profiler array. See Supplementary_Table_1
