# Supplementary material for: Silencing of casein kinase 1 delta reduces migration and metastasis of triple negative breast cancer cells
Source: Oncotarget. 2018 Jul 20;9(56):30821–36. doi: 10.18632/oncotarget.25738 (PMC6089398; doi:10.18632/oncotarget.25738)
Supplement: Supplementary file 2 [file oncotarget-09-30821-s002.pdf]

Gene Symbol	AVG ΔC_t		2^-ΔC_t		Fold Change	Fold Up- or Down-Regulation
	shCSNK1D	shNT	shCSNK1D	shNT	shCSNK1D/shNT	shCSNK1D/shNT
AHNAK	5.91	6.24	0.016617	0.013237	1.26	1.26
AKT1	6.56	5.75	0.010590	0.018590	0.57	-1.76
BMP1	10.49	10.13	0.000695	0.000893	0.78	-1.29
BMP2	13.93	13.07	0.000064	0.000116	0.55	-1.82
BMP7	13.93	13.07	0.000064	0.000116	0.55	-1.82
CALD1	4.13	4.35	0.057066	0.049060	1.16	1.16
CAMK2N1	4.21	3.29	0.053988	0.102286	0.53	-1.89
CAV2	5.46	5.86	0.022699	0.017225	1.32	1.32
CDH2	12.64	11.64	0.000157	0.000313	0.50	-2.00
COL1A2	13.93	13.07	0.000064	0.000116	0.55	-1.82
COL3A1	13.78	13.07	0.000071	0.000116	0.61	-1.64
COL5A2	9.12	9.59	0.001796	0.001298	1.38	1.38
CTNNB1	6.98	7.09	0.007915	0.007343	1.08	1.08
DSC2	10.15	10.12	0.000879	0.000899	0.98	-1.02
DSP	4.48	4.22	0.044773	0.053686	0.83	-1.20
EGFR	4.24	4.13	0.052877	0.057141	0.93	-1.08
ERBB3	9.16	9.83	0.001747	0.001099	1.59	1.59
ESR1	12.67	12.21	0.000153	0.000211	0.73	-1.38
F11R	8.49	8.94	0.002779	0.002037	1.36	1.36
FGFBP1	12.12	12.94	0.000224	0.000127	1.76	1.76
FN1	2.92	3.19	0.132016	0.109628	1.20	1.20
FOXC2	13.61	12.34	0.000080	0.000193	0.41	-2.41
FZD7	10.89	10.75	0.000527	0.000581	0.91	-1.10
GNG11	9.54	8.67	0.001342	0.002456	0.55	-1.83
GSC	13.93	13.07	0.000064	0.000116	0.55	-1.82
GSK3B	4.83	4.65	0.035128	0.039849	0.88	-1.13
IGFBP4	8.02	8.53	0.003849	0.002707	1.42	1.42
IL1RN	12.65	13.07	0.000155	0.000116	1.34	1.34
ILK	3.73	3.68	0.075299	0.078058	0.96	-1.04
ITGA5	6.83	6.77	0.008782	0.009167	0.96	-1.04
ITGAV	5.72	6.19	0.018956	0.013703	1.38	1.38
ITGB1	0.81	0.58	0.569900	0.669280	0.85	-1.17
JAG1	4.58	3.66	0.041775	0.079147	0.53	-1.89
KRT14	12.93	11.07	0.000128	0.000465	0.28	-3.63
KRT19	2.81	2.75	0.142475	0.148721	0.96	-1.04
KRT7	3.69	4.21	0.077416	0.054059	1.43	1.43
MAP1B	1.75	1.37	0.297050	0.387074	0.77	-1.30
MMP2	13.93	13.07	0.000064	0.000116	0.55	-1.82
MMP3	8.41	7.55	0.002937	0.005339	0.55	-1.82
MMP9	12.71	13.01	0.000149	0.000121	1.23	1.23
MSN	1.75	2.18	0.297050	0.220780	1.35	1.35
MST1R	12.47	11.90	0.000176	0.000262	0.67	-1.49
NODAL	12.51	13.07	0.000171	0.000116	1.47	1.47
NOTCH1	12.62	12.28	0.000159	0.000201	0.79	-1.27
NUDT13	8.37	8.21	0.003020	0.003379	0.89	-1.12
OCLN	4.28	4.78	0.051431	0.036415	1.41	1.41

PDGFRB	13.93	13.07	0.000064	0.000116	0.55	-1.82
PLEK2	5.73	5.14	0.018825	0.028373	0.66	-1.51
DESI1	3.87	3.20	0.068336	0.108870	0.63	-1.59
PTK2	4.62	4.71	0.040633	0.038226	1.06	1.06
PTP4A1	2.59	2.29	0.165945	0.204572	0.81	-1.23
RAC1	1.04	0.61	0.485916	0.655507	0.74	-1.35
RGS2	6.48	5.99	0.011193	0.015741	0.71	-1.41
SERPINE1	5.58	5.79	0.020887	0.018082	1.16	1.16
GEMIN2	4.36	3.85	0.048657	0.069381	0.70	-1.43
SMAD2	5.64	5.32	0.020037	0.025045	0.80	-1.25
SNAI1	13.93	13.07	0.000064	0.000116	0.55	-1.82
SNAI2	3.93	3.08	0.065552	0.118313	0.55	-1.80
SNAI3	10.79	10.44	0.000564	0.000720	0.78	-1.28
SOX10	13.93	13.07	0.000064	0.000116	0.55	-1.82
SPARC	13.93	13.07	0.000064	0.000116	0.55	-1.82
SPP1	13.93	11.76	0.000064	0.000288	0.22	-4.51
STAT3	3.71	3.60	0.076350	0.082508	0.93	-1.08
STEAP1	2.74	2.65	0.149558	0.159395	0.94	-1.07
TCF3	3.23	3.08	0.106489	0.118313	0.90	-1.11
TCF4	8.89	8.62	0.002106	0.002543	0.83	-1.21
TFPI2	3.96	2.94	0.064203	0.130370	0.49	-2.03
TGFB1	7.51	7.21	0.005481	0.006757	0.81	-1.23
TGFB2	4.83	3.15	0.035128	0.112710	0.31	-3.21
TGFB3	9.74	10.28	0.001168	0.000805	1.45	1.45
TIMP1	6.16	5.89	0.013973	0.016871	0.83	-1.21
TMEFF1	5.45	5.06	0.022857	0.029991	0.76	-1.31
TMEM132A	12.98	13.07	0.000124	0.000116	1.06	1.06
TSPAN13	8.08	7.54	0.003692	0.005376	0.69	-1.46
TWIST1	13.93	13.07	0.000064	0.000116	0.55	-1.82
VCAN	9.75	9.81	0.001160	0.001115	1.04	1.04
VIM	-3.11	-3.66	8.626.526	12.646.638	0.68	-1.47
VPS13A	4.64	4.05	0.040073	0.060400	0.66	-1.51
WNT11	13.93	13.07	0.000064	0.000116	0.55	-1.82
WNT5A	11.45	10.12	0.000357	0.000899	0.40	-2.52
WNT5B	8.88	8.37	0.002121	0.003024	0.70	-1.43
ZEB1	6.61	5.96	0.010229	0.016072	0.64	-1.57
ZEB2	5.97	6.35	0.015940	0.012265	1.30	1.30

Supplementary table 1 : RT2 profiler array . The results are calculated using delta delta CT method, in which delta CT is calculated between gene of interest (GOI) and an average of reference genes (ACTB B2M, GAPDH, HPRT1 and RPLP0), followed by delta-delta CT calculations (delta CT (Test Group)-delta CT (Control Group)). Fold Change is then calculated using 2^ (-delta delta CT) formula .
